# Upgrading Recycled Polypropylene from Textile Wastes in Wood Plastic Composites with Short Hemp Fiber

**DOI:** 10.3390/polym13081248

**Published:** 2021-04-12

**Authors:** Francisco Burgada, Eduardo Fages, Luis Quiles-Carrillo, Diego Lascano, Juan Ivorra-Martinez, Marina P. Arrieta, Octavio Fenollar

**Affiliations:** 1 Textile Research Institute (AITEX), Plaza Emilio Sala 1, 03801 Alcoy, Spain; fburgada@aitex.es (F.B.); efages@aitex.es (E.F.); 2 Technological Institute of Materials (ITM), Universitat Politècnica de València (UPV), Plaza Ferrándiz y Carbonell 1, 03801 Alcoy, Spain; luiquic1@epsa.upv.es (L.Q.-C.); dielas@epsa.upv.es (D.L.); juaivmar@doctor.upv.es (J.I.-M.); 3 Escuela Politécnica Nacional, Quito 170517, Ecuador; 4 Departamento de Ingeniería Química Industrial y del Medio Ambiente, Escuela Técnica Superior de Ingenieros Industriales, Universidad Politécnica de Madrid (ETSII-UPM), C/ José Gutiérrez Abascal 2, 28006 Madrid, Spain; m.arrieta@upm.es; 5 Grupo de Investigación: Polímeros, Caracterización y Aplicaciones (POLCA), 28006 Madrid, Spain

**Keywords:** recycled polypropylene, short hemp fiber, injection molding, compatibilization, polymer composites

## Abstract

This research reports the manufacturing and characterization of green composites made from recycled polypropylene obtained from the remnants of polypropylene non-woven fabrics used in the textile industry and further reinforced with short hemp fibers (SHFs). To improve the interaction of the reinforcing fibers with the recycled polymeric matrix, two types of compatibilizing agents (maleic anhydride grafted, PP-g-MA, and maleinized linseed oil, MLO) were added during melt-processing, the percentage of which had to remain constant concerning the amount of fiber loading to ensure complete reactivity. Standardized test specimens were obtained by injection molding. The composites were characterized by mechanical (tensile, impact, and hardness), thermal (DSC, TGA), thermomechanical, FTIR, and FESEM microscopy tests. In addition, color and water uptake properties were also analyzed. The results show that the addition of PP-g-MA to rPP was satisfactory, thus improving the fiber-matrix interaction, resulting in a marked reinforcing effect of the hemp fibers in the recycled PP matrix, which can be reflected in the increased stiffness of the samples. In parallel to the compatibilizing effect, a plasticizing effect was obtained by incorporating MLO, causing a decrease in the glass transition temperature of the composites by approximately 6 °C and an increase in ductility compared to the unfilled recycled polypropylene samples.

## 1. Introduction

New materials research over the last 40 years has led to an increase in the number of wood plastic composites (WPCs). Currently, they can be used in many applications such as decking (indoor or garden furniture), packaging, or consumer goods [1,2]. The automotive industry is one of the areas where these composites have a great incursion due to their excellent weight-strength ratio. The use of natural fibers in polymeric matrices could reduce about 20% of the overall weight and 30% of the cost. Weight reduction in cars leads to fuel saving and as a result a reduction in greenhouse gases [3]. Natural fibers, such as hemp, flax, and jute, among others, are used as a reinforcement in thermoplastics due to their good mechanical properties and their ease of acquisition. This factor facilitates the replacement of synthetic fibers such as aramid, carbon, and glass currently used because the production of synthetic fibers is more expensive, and they have a negative effect on the environment [4,5]. Moreover, the use of bio-based materials with the aim to reduce the dependency on petroleum-based materials is an actual trend [6]. This trend is related to the principles proposed by the circular economy, which seeks to reuse or recycle wastes [7]. In this context, wool fibers obtained from industrial wastes are gaining considerable interest as plastics-reinforcing fillers [8,9]. Hemp fibers come from the *Cannabis Sativa* plant family, an annual plant that grows in temperate regions like China. The main properties of hemp are good mechanical strength and Young modulus; cellulose content is between 55% and 72%, and lignin content is around 2–5% [10,11]. Hemp fibers are mainly used for textile applications. However, a bundle of short hemp fibers (SHFs) is usually excluded from textile use as a by-product mainly because of their poor resistance [12]. Therefore, WPCs based on SHF have gained considerable interest in several industrial sectors, such as automotive, building, and furnishing industries.

Annually, between 25 and 30 million tons of plastic waste are generated in Europe (in 2018, 29.1 million tons were generated [13]), and only about 30% can finally be recycled [13,14]. Polyolefins (polyethylene and polypropylene) are the most demanded plastic resins by industrial converters. Particularly, PP is highly demanded for several industrial sectors. Thus, one of the main components from the generated waste is polypropylene (PP) since it is employed in many applications because of its interesting properties such as low density, good processability, and a competitive price. Some of the applications can be injection molded parts for the automobile industry or household appliances, as well as single-use plastic products [15,16]. According to Gu et al. [17], recycling plastics could save 20–50% of the cost compared to using virgin materials. Other benefits of recycling thermoplastics are the reduction of pollution produced when they are incinerated and the reduction of the volume of waste in the landfill [15], as well as the reduction of petrochemical sources consumption for the production of virgin PP.

Nowadays, synthetic fibers account for about two-thirds of the fiber production every year, which consumes about 14.5% of the global plastic production. For this reason, part of this plastic waste comes from the textile industry where the production of synthetic fiber increased by 15% between 2017 and 2018 [18]. Following the recommendations proposed by the European Parliament in the Waste Framework Directive, if the textile cannot be used, it should be recycled as proposed in the 2008/98/EC directive [19]. There are some applications where recycled polypropylene can be employed. For instance, Yin et al. [20] proposed the introduction of recycled fibers as a reinforcement of concrete; they compared the difference between virgin fibers, an actual alternative to steel meshes in concrete. Similar results were obtained for both kinds of fibers, therefore they can replace the steel meshes.

As there is a huge amount of recycled thermoplastics, they can be a promising raw source for WPCs, especially because of the low cost of these kinds of materials [21]. However, wood fibers traditionally reported hydrophilic nature promoted by the hydroxyl groups in cellulose, hemicellulose, and lignin, which represents one of the main drawbacks of natural fibers as reinforcing fillers of thermoplastic polymers [22]. This behavior results in a lack of compatibility between the polymeric matrix and the fibers. Poor adhesion between the fibers and the polymeric matrix could promote water absorption and also a decrease in the mechanical properties [23,24], consequently leading to the deterioration of the properties during the aging of the WPC product. Thus, the high hydrophilic nature of wood fibers limits the WPC outdoor applications, such as windows, frames, furniture, decking, and construction materials [25]. Another important disadvantage of natural fibers for WPC production is their thermal sensitiveness since the fibers can be degraded either during composite thermal processing and/or during composite material service [26]. To this effect, there are several methods to improve the polymer/ natural fiber interaction, among which are the incorporation of nanoparticles or a silane treatment to the fibers, another possibility is to introduce compatibilizers or coupling agents based on maleic anhydride (MA) that promote the hydrogen and covalent bonds with hydroxyl groups of the cellulose [27,28]. Although PP has been widely reinforced with natural fibers compatibilized with PP-g-MA, recycled PP (rPP) WPCs have been less studied. In this context, Islam et al. reinforced rPP with kenaf fibers using PP-g-MA as compatibilizer and observed a significant reduction of the water absorption of rPP/kenaf fiber due to the PP-g-MA addition [29]. Srebrenkoska et al. prepared PP reinforced with kenaf fiber composites compatibilized with PP-g-MA and further simulated the recycling process by reprocessing the composites; they observed that recycled composites showed some improvement on the interfacial adhesion [30]. Kord et al. reinforced virgin PP with 50 wt% of hemp fibers (100 mm in length) compatibilized with PP-g-MA and compared it with rPP-based composites obtaining the rPP from the waste spindle during the processing of virgin PP. The rPP-based composites showed a higher swelling effect than virgin PP-based composites due to the high hydrophilicity of hemp fiber and required a fourth component such as a clay (montmorillonite) to reduce the hydrophilicity of the composites [31]. On the other hand, some authors reported that the introduction of maleinized linseed oil (MLO) enhanced the compatibility of different WPCs by the formation of bonds between the carboxylic ester of MLO and the hydroxyls groups of cellulose [32,33]. Nevertheless, MLO compatibilized SHFs as the reinforcing phase for PP and/or rPP has not been studied yet.

With the main objective of mitigating the environmental problems associated with polypropylene textile wastes, in this work, the reuse of polypropylene fiber industrial wastes in the manufacture of wood plastic composites was evaluated. The rPP was obtained from non-woven polypropylene fabrics, and composites were prepared by an injection molding process to simulate the most typical WPC industrial processing conditions. The influence of short hemp fibers (SHFs), another textile waste, as reinforcement fiber for rPP, was studied by loading rPP with 30 wt% of SHFs (with length less than 3 mm). Since the main drawback of natural fibers is the high hydrophilicity which leads to low compatibility with PP matrix, two types of compatibilizing agents were used, polypropylene grafting maleic copolymer (PP-g-MA) and maleinized linseed oil (MLO), to improve the interaction between the reinforcement fibers and the polymeric matrix. The wood plastic composites were evaluated in terms of mechanical, thermal, and thermo-mechanical properties. The interaction between the matrix and the reinforcing fiber was analyzed by FTIR and FESEM. Finally, since these composites are intended for industrial applications where the hydrophobicity represents a handicap (i.e., automotive, construction materials, outdoor applications, etc.), the effect of the addition of compatibilized short hemp fibers in different percentages (10, 20, and 30 wt%) on the water absorption properties of the rPP-based composites was studied. Finally, the composites’ appearance was evaluated, and the color properties were assayed to get insights regarding the industrial applicability of the PP textile wastes recyclability as WPC materials.

## 2. Materials and Methods

### 2.1. Materials

**Figure 1:**
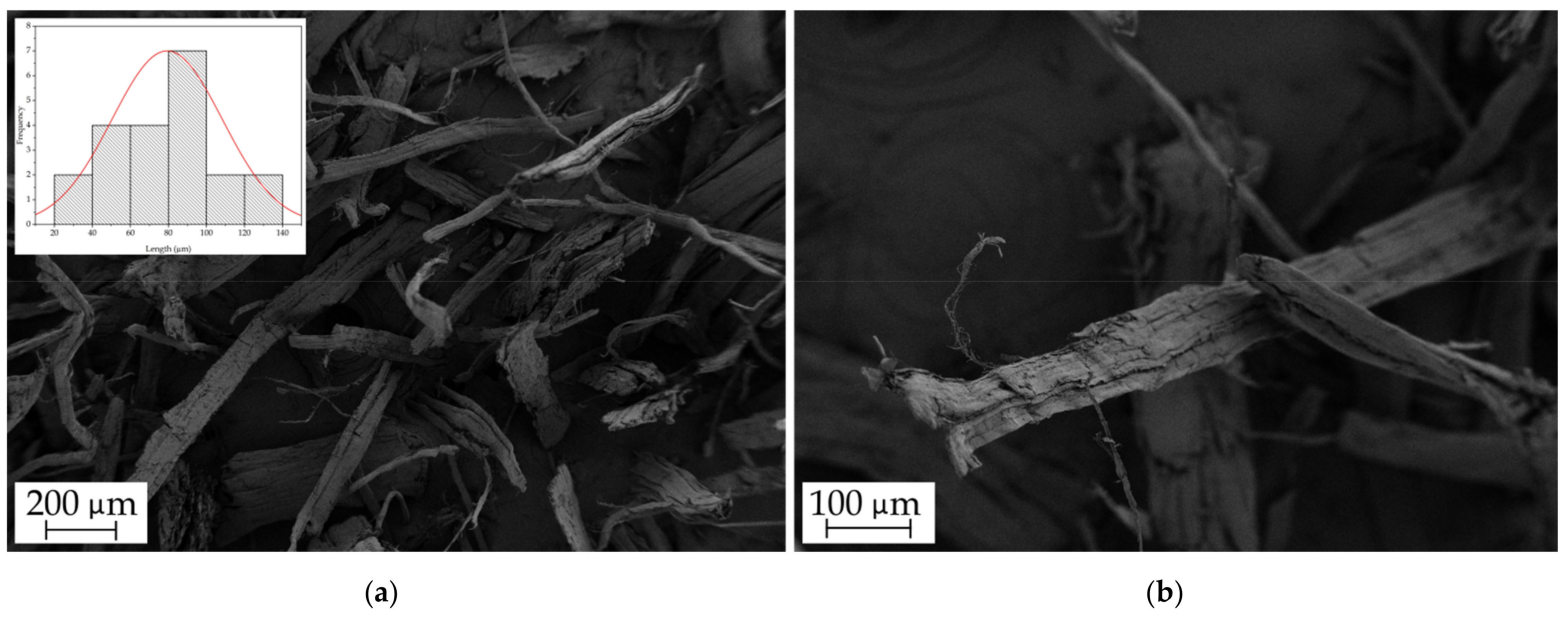
FESEM images of short hemp fiber at (**a**) 50× with a scale marker of 200 µm with a fiber fineness histogram and (**b**) 125× with a scale marker of 100 µm.

A recycled polypropylene (rPP) was used as a matrix of the manufacture of the compounds, this material was obtained from the unused pieces of the ends of the non-woven polypropylene fabrics supplied by AITEX (Alcoy, Spain). A short hemp fiber (SHF) type F 517 supplied by Schwarzwälder Textil-Werke Heinrich Kautzmann GmbH (Schenkenzell, Germany) was used as a fiber filler; it has an approximate fiber coarseness of 50–80 µm as can be seen in Figure 1, and it has a specific gravity of 1.48–1.50 g cm^−3^. It also presents an approximate tensile strength of ≈1100 N mm^−2^.

Two types of compatibilizers were used to improve the interaction between the matrix and the reinforcing fibers, a commercial maleic anhydride grafted, metallocene-catalyzed polypropylene wax grade Licocene PP MA 6452 granules from Clariant Plastics & Coatings (Frankfurt, Germany) (PP-g-MA), and a maleinized linseed oil (MLO) from VEOMER LIN by Vandeputte (Mouscron, Belgium) with a viscosity of 10 dPa s (20 °C).

### 2.2. Manufacturing of the Recycled Polypropylene Sample and the rPP/Hemp Fiber Composites

To avoid later defects in the samples caused by moisture, the materials were subjected to a drying stage at 60 °C overnight before processing. All formulations were mixed in the first stage by manual methods in a ziplock bag to obtain uniform mixtures. Table 1 shows the coding and formulations of the compounds manufactured. The formulations are designed so that the amount of compatibilizer remains constant with increasing load. For instance, for every 10% load, 2.5 phr is added; they were extruded in a twin-screw co-rotating extruder from Dupra S.L. (Alicante, Spain). The temperature profile was selected considering the melting peak temperature of a conventional polypropylene (PP), starting at the feed hopper with 155 °C and ending at the nozzle with 175 °C, and the rotating speed was set at 20 rpm. Once the strands were obtained, they were cooled down to room temperature in air, and then pelletized in an air knife unit. To obtain standard specimens, the pellets from all formulations were then injection-molded in an injection molding machine model Meteor 207/75 from Mateur & Solé (Barcelona, Spain). The temperature profile was 155 °C in the feed hopper and increased by 5 degrees at each stage, up to the injection nozzle with 170 °C; the injection and cooling times were set at 1 and 10 s, respectively.

### 2.3. Mechanical Properties

Tensile test of recycled polypropylene sample and the rPP/ hemp fiber composites was carried out on a universal testing machine ELIB 30 from Ibertest (Madrid, Spain); test parameters and dog-bone shape specimen were according to ISO 527 standard. The machine was equipped with a 5 kN load cell with a crosshead rate of 10 mm min^−1^. Through this test, the tensile modulus, E_t_, tensile strength, σ_b_, and elongation at break, ε_b_, were obtained. The impact strength of the samples was determined on a Charpy pendulum from Metrotec S.A. (San Sebastian, Spain); this was done on unnotched samples using a 1 J pendulum according to ISO 179 standard. Five specimens of each formulation were tested to obtain reliable data. In addition, the Shore D hardness was measured using a durometer model 637-D from J. Bot S.A. (Barcelona, Spain), following the guidelines of ISO 868 standard, with a stabilization time of 15 s. Measured at five different points and averaged, this process was repeated for all formulations.

### 2.4. Morphological Characterization

The morphological study of the fractured surfaces of the samples subjected to impact tests was carried out by field-emission scanning electron microscopy (FESEM). A Zeiss Ultra 55 FESEM microscope supplied by Oxford Instruments (Abingdon, UK) was used. An electron acceleration voltage of 1.5 kV was established. Prior to the study, the sample surfaces were subjected to a sputtering coating process using an ultrathin gold-palladium layer in a high vacuum sputter coater model EM MED20 supplied by Leica Microsystem (Milton Keynes, UK).

### 2.5. Thermal Properties

The determination of the main thermal transitions of recycled polypropylene sample and the rPP/hemp fiber composites’ compounds was carried out by differential scanning calorimetry (DSC) using an 821 DSC calorimeter supplied by Mettler-Toledo Inc. (Schwerzenbach, Switzerland). This test was performed with samples weighing approximately 5 mg, which were subjected to a three-stage thermal cycle. The first stage consists of a heating process from room temperature to 180 °C in order to erase the thermal history caused to the previous processing stages, followed by a cooling stage down to −30 °C. Finally, a second heating cycle from −30 °C to 200 °C. Both the heating stages (first and second cycle) and the cooling cycle were programmed at a constant heating and cooling rate of 10 °C min^−1^, respectively. Tests were conducted under an inert atmosphere to prevent unwanted oxidation, and thus a constant flow of nitrogen (66 mL min^−1^) was maintained throughout the test. The crystallization temperature (*T_c_*), melting enthalpy (Δ*H_m_*), and the peak melting temperature (*T_m_*) could be obtained from the cooling and the second heating cycle, respectively. The maximum crystallinity, *χ_c___max_*, of the different compounds can be obtained by Equation (1).

(1) $$\chi_{c\_max}=\frac{\Delta H_{m}}{\Delta H_{m}^{0}\cdot \left(1-w\right)}\cdot 100\;\%$$

where $\Delta H_{m}^{0}$ correspond to the theoretical melting enthalpy of fully crystalline polypropylene, which according to the literature, is 138 J g^−1^ [34,35], and w is the filler mass fraction.

The thermal stability and thermal degradation of recycled polypropylene sample, the short hemp fibers, and the rPP/hemp fiber composites were studied by thermogravimetric analysis (TGA) using a thermobalance model TGA1000 from Linseis (Selb, Germany). The average weight of the samples was between 10 and 20 mg. These were placed in standard alumina crucibles (70 mL) to obtain comparable data; the weight of the samples was kept constant. All samples were exposed to a dynamic heating cycle from 30 up to 700 °C at a constant heating rate of 10 °C min^−1^ under inert atmosphere, and thus a constant flow of nitrogen of 66 mL min^−1^ was used during the tests. Thermal parameters such as the onset degradation temperature, which is assumed to occur when the sample loses 5% of its mass, *T_5%_*, and maximum degradation rate temperature, *T_deg_*, could be obtained from this test.

### 2.6. Thermomechanical Properties

Dynamic mechanical behavior of the recycled polypropylene sample and the rPP/hemp fiber composites was analyzed by a dynamic mechanical thermal analysis (DMTA) in a DMA1 dynamic analyzer from Mettler-Toledo (Schwerzenbach, Switzerland); the equipment operates in single cantilever/flexural conditions. Solid samples with dimensions of 20 × 7 × 1 mm were used and subjected to a temperature sweep from −100 to 120 °C at a constant heating rate of 2 °C min^−1^, with a frequency of 1 Hz and maximum flexural deformation (%γ) of 0.1%. The test allowed us to observe the evolution of the storage modulus, *E*’, and the dynamic damping factor, tan *δ*, as a function of the temperature, allowing the determination of thermal transition such as the glass transition temperature, *T_g_*.

### 2.7. Color Measurements

The influence of short hemp fiber addition on the color of rPP/hemp fiber compounds was studied in a colorimeter KONICA CM-3600d Colorflex-DIFF2 from Hunter Associates Laboratory (Reston, VA, USA). The CIELab color scale was used to determine the L*, a*, and b* coordinates, where L* represents the luminance, a* shows the change between the red and green color, and b* shows the change between the yellow and blue color. The equipment was calibrated taking into account the standard white tile and a mirror device for the black. The total color difference, $\Delta E_{ab}^{*}$, was obtained following Equation (2).

(2) $$\Delta E_{ab}^{*}=\sqrt{{\left(\Delta L^{*}\right)}^{2}+{\left(\Delta a^{*}\right)}^{2}+{\left(\Delta b^{*}\right)}^{2}}$$

where $\Delta L^{*}$, $\Delta a^{*},\;\mathrm{and}\;\Delta b^{*}$ are the differences between the color of the samples and the reference color.

### 2.8. Water Absorption Analysis

Water absorption of the recycled polypropylene sample and the rPP/hemp fiber composites was performed following the guidelines of ISO 62:2008 standard. The test consists of immersing samples (80 × 10 × 4 mm) of the different formulations in distilled water at room temperature for a period of 15 weeks. The samples were removed from the water, dried, and then weighed on an analytical balance AG245 from Mettler-Toledo (Schwerzenbach, Switzerland) with an accuracy of ±0.001 g, then finally immersed again in the water. This process was carried out once a week during the experimental period to ensure the veracity of the results, and all measurements were made in triplicate. The percentage of water absorption was calculated with the following Equation (3).

(3) $$Water\;absorption\;\left(\%\right)=\frac{\left(W_{t}-W_{0}\right)}{W_{0}}\;\cdot 100$$

where *W_t_* is the weight of the dry sample in a time *t* and *W_0_* is the weight of the initial dry sample.

### 2.9. Chemical Structure and Infrared Spectroscopy

A chemical analysis of the rPP/SHF green composites was carried out by attenuated total reflection-Fourier-transform infrared (ATR-FTIR) spectroscopy. Spectra were recorded using a Bruker S.A Vector 22 (Madrid, Spain) coupled to a PIKE MIRacleTM single reflection diamond ATR accessory (Madison, Wisconsin, USA). Data were collected as the average of 15 scans between 4000 and 650 cm^−1^ with a spectral resolution of 4 cm^−1^.

## 3. Results

### 3.1. Mechanical Properties of the Recycled Polypropylene Sample and the rPP/Hemp Fiber Composites

The results obtained by the mechanical characterization process are shown in Table 2 (tensile test, hardness, and impact Charpy) of rPP sample and rPP/SHF composites. The incorporation of short hemp fibers leads to an improvement of the elastic modulus. The addition of 10 wt% SHF results in an increase in stiffness to 1617 MPa from 933 MPa (compared with neat rPP). The elastic modulus shifted up to 2977 MPa for the 30 wt% compatibilized with PP-g-MA, which means about 320% more than the neat rPP. The effect of the compatibilizer (PP-g-MA) is clearly observed in the rPP-H30 and the rPP-H30-C samples, improving the interaction between the thermoplastic matrix and the lignocellulosic fiber, the elastic modulus increased from 2339 MPa to 2977 MPa (27.2% more), and similar results were reported by Sullins et al. [36]. The use of MLO results in a different effect on the rPP/hemp composites. In fact, the elastic modulus of rPP-H30-M decreased.

The use of MLO with a lignocellulosic filler could produce different phenomena like the plasticization effect at the same time as the compatibilization effect due to an interaction of hydroxyl groups in cellulose (SHF) and the polar groups of MLO [37,38]. The samples compatibilized with PP-g-MA showed an increasing tensile strength by the addition of hemp fibers, reaching values of 28.9 MPa for the rPP-H30-C samples approximately 32% more than neat rPP (21.8 MPa). The absence of compatibilizer produced that the rPP-H30 had a tensile strength of 22.4 MPa, only 5.6% more than neat rPP. This is due to the low interaction of the fibers with the matrix, causing voids between the matrix and the fiber that act as stress concentrators [39]. rPP-30-M samples registered a tensile strength of 17.7 MPa, 16.5% less than neat rPP, as Liminana et al. [38], suggesting that MLO reduced the resistant properties in PBS/ASF composites. The elongation at break (εb) is highly affected by the introduction of short fiber in the polymeric matrix; the neat rPP obtained an elongation at break of 100%, and the addition of 10% of SFH led to a reduction of 93.3%. Higher contents of hemp slightly changed the elongation at break of the compatibilized composites. As was mention above, the addition of MLO provided a plasticizing effect, which results in an improvement of the elongation at break compared with the H30-C samples.

The incorporation of microfibers also has an effect on the hardness of the different composites. This effect could be seen in greater depth for rPP-H30-C, which obtained values of 76 on the Shore D hardness scale, 8.1% more than neat rPP. According to Ivorra-Martinez et al. [40], hardness follows a trend similar to that of stiffness in composite materials. It can be observed that the use of MLO results in materials with a lower hardness, being even lower than that of rPP. The improvement of impact strength properties by introducing fibers into polymeric matrices has been reported by many authors previously. This effect can be attributed to the ability of the materials to dissipate energy by pulling the fibers away from the thermoplastic matrix [10,11]. In fact, the improvement of the impact strength properties could be seen by adding 20 wt% SFH content, with an improvement of 9.1% compared to neat rPP. The dissipation of energy during the debonding of the fibers is highly affected by the interaction between the fiber and the matrix, for this reason, rPP-H30 values were 15.6% lower than the one compatibilized with PP-g-MA [41,42]. Moreover, the use of MLO provided an extra improvement by the plasticization. Quiles et al. [43] reported similar results when they introduced AESO (acrylate epoxidized soybean oil) into PLA.

### 3.2. Morphological Characterization of the Recycled Polypropylene Sample and the rPP/Hemp Fiber Composites

**Figure 2:**
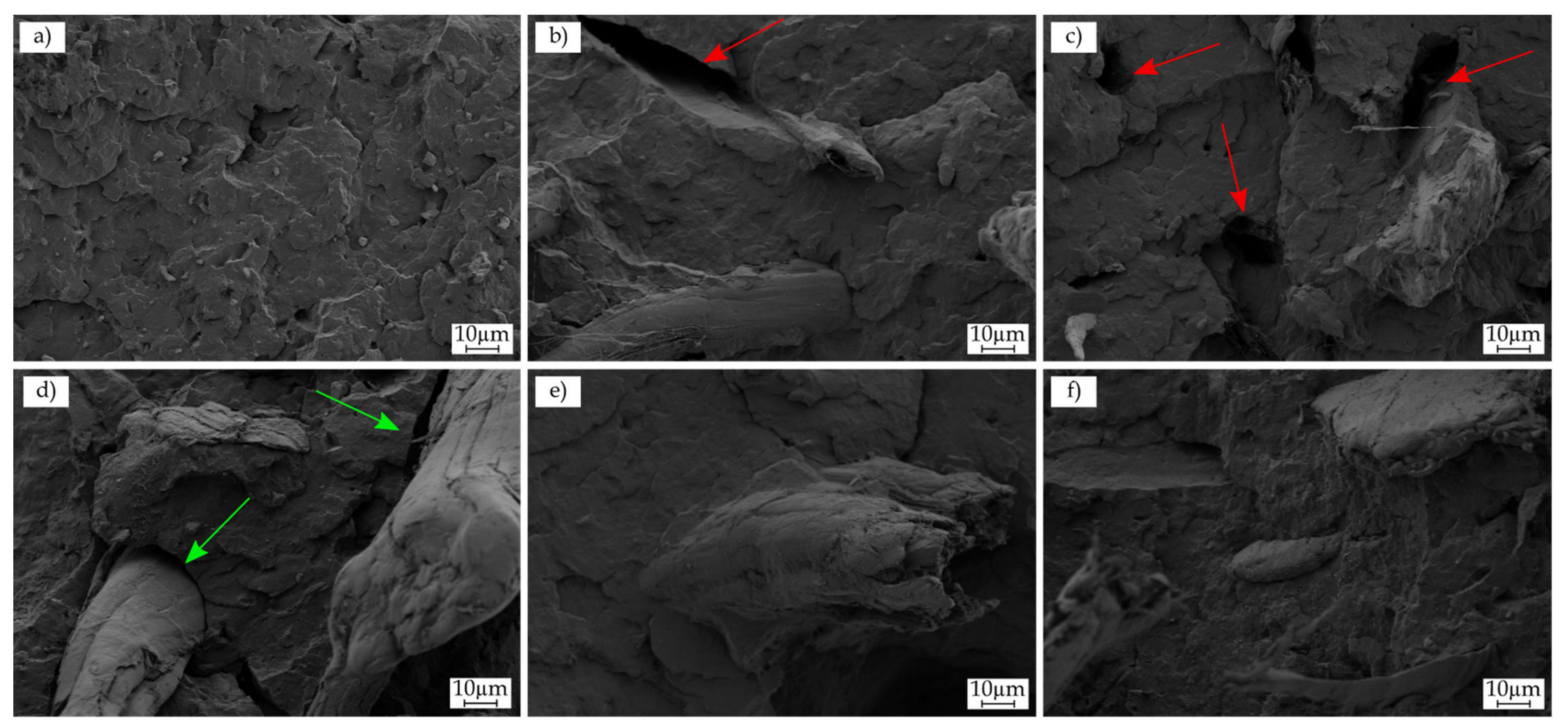
Field-emission scanning electron microscopy (FESEM) images of the fracture surfaces of the injection-molded rPP/hemp composite parts: (**a**) rPP; (**b**) rPP-H10-C; (**c**) rPP-H20-C; (**d**) rPP-H30; (**e**) rPP-H30-C; and (**f**) rPP-H30-M. Images were taken at 500×.

To assess the quality of the rPP polymeric matrix/fiber interface, the morphology of the fracture surfaces of the injection-molded samples after the impact tests was observed by FESEM, and the images are shown in Figure 2. The fracture surface of the rPP sample (Figure 2a) shows roughness, and this phenomenon is related to the plastic deformation of the thermoplastic. One feature of this rPP is the presence of some particles inside the polymer matrix, and this phenomenon was attributed to the recycling process wherein these particles have been mixed. The incorporation of SHF does not show a significant effect on fracture roughness, but a somewhat reduced roughness can be detected. On the other hand, the addition of MLO increased the roughness due to the plasticization of the rPP matrix that promoted the ability of plastic deformation (Figure 2f).

As proposed in the mechanical characterization, a debonding phenomenon of the fibers was produced when the composites were subjected to the impact test. Some holes produced by the fibers that were pulled out during the breakage are remarked with red arrows [44,45]. Additionally, the effect of the compatibilizer could be seen in the non-compatibilized rPP (Figure 2d). The green arrows show the gaps between the SHF and rPP matrix confirming the poor phase adhesion produced by the lack of compatibility between the fiber and the polymeric matrix. The incorporation of the different compatibilizers helped to reduce this effect as the gaps between rPP and compatibilized hemp fibers mainly disappeared (Figure 2e,f). The morphologies observed, corroborate the mechanical properties obtained in terms of the compatibilizer employed. The absence of the compatibilizer promoted a deterioration of the resulting values as a result of the gap formation between the thermoplastic and the hemp [40]. Meanwhile, the compatibilization of SHF with both PP-g-MA and MLO promotes a more effective fiber/polymeric matrix adhesion as can be clearly seen in Figure 2e,f, respectively.

### 3.3. Thermal Properties of the Recycled Polypropylene Sample and the rPP/Hemp Fiber Composites

**Figure 3:**
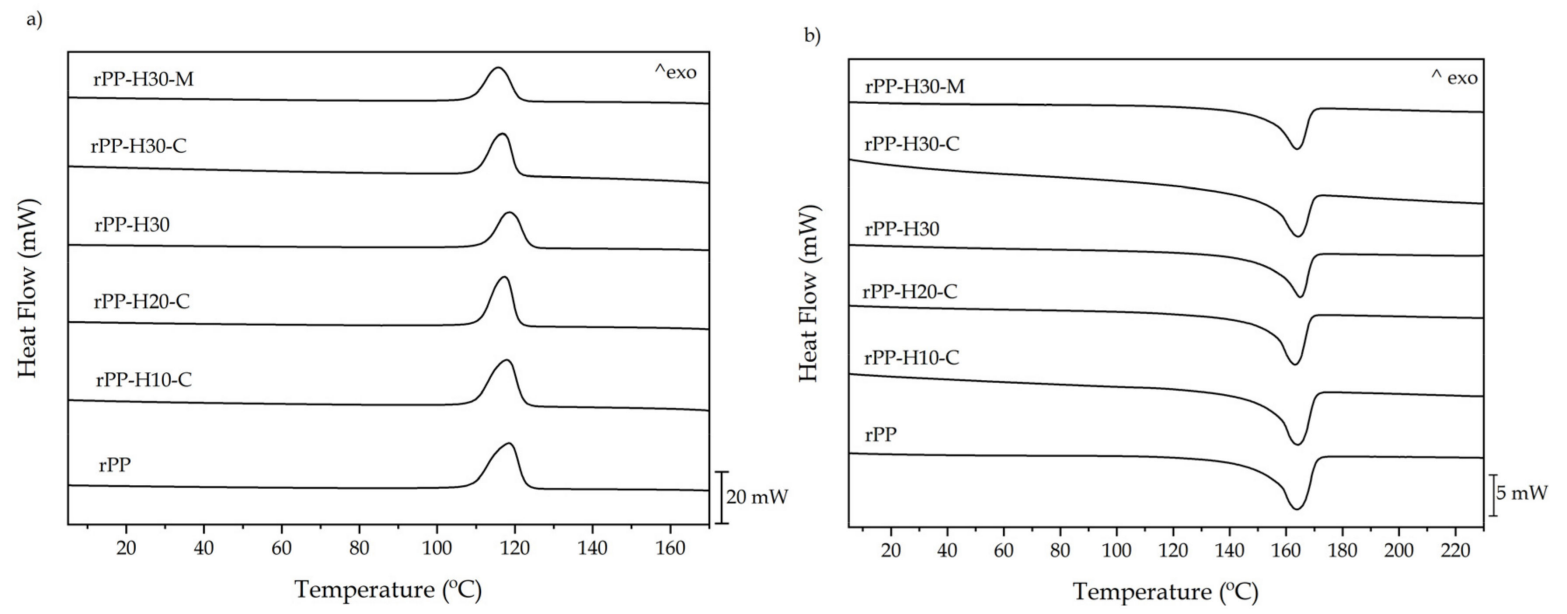
DSC thermograms of rPP/hemp fiber composites’ (**a**) cooling cycle and (**b**) second heating cycle.

The main thermal transitions of recycled polypropylene sample and the rPP/hemp fiber composites were obtained by the DSC analysis. Figure 3 displayed the comparative thermograms of the compounds. Figure 3a shows the cooling cycle; from this cycle, it was possible to record the crystallization temperature (*T_c_*). The rPP has a crystallization peak at around 118.34 °C. Luyt et al. [46] showed very similar results for virgin polypropylene, with a value of 115 °C. The melting point (*T_m_*) and the melting enthalpy (Δ*H_m_*) were extracted from the second heating cycle (Figure 3b). Table 3 gathers the main parameters extracted from the thermograms.

Regarding the melting point (*T_m_*), it is observed that the recycled polypropylene unfilled presents a value of 163.7 °C, which is a value similar to that of virgin polypropylene [34] as well as recycled polypropylene [47], according to the literature. When a low amount of hemp fiber (i.e., 10% and 20%) is incorporated with PP-g-MA compatibilizer, no noticeable variation in melting temperature is observed; an increase of about 2 °C can be seen with the addition of 30% hemp fiber. It should be noted that the addition of both compatibilizers (PP-g-MA or MLO) to the structure does not influence the melting temperature of the composite materials, maintaining a temperature similar to that of unfilled recycled polypropylene (163 °C). The peak of the crystallization temperature decreases slightly with the incorporation of 10%, 20%, and 30% hemp fiber with PP-g-MA. This refers to the good interaction of the reinforcing fibers with the matrix due to the strong bonding generated by reacting the maleic anhydride groups of PP-g-MA with the hydroxyl groups of the hemp fibers; good bonding with the PP chains also occurs, resulting in grafted maleic anhydride copolymers [48]. It is worth noting that the use of MLO as a compatibilizer when incorporating 30% of hemp fiber results in a decrease in *T_c_* reaching values of 115.7 °C. This reduction may be due to the fact that the MLO in addition to the compatibilizing effect causes a plasticizing effect in the rPP matrix, and this leads to greater mobility of the polymeric chains, and consequently, the interaction between them somewhat decreases [49,50]. This could also lead to a decrease in the contact between the reinforcement fibers and the matrix, causing the nucleating effect of these compatibilized fibers on the rPP matrix tent to decrease [51]. Concerning crystallinity, the recycled polypropylene without reinforcement fibers presents a percentage close to 68.3%. These results are similar to the ones presented by Garcia-Garcia et al. [52] and correspond to virgin polypropylene. It is observed that the addition of 20% of hemp fiber considerably increases the crystallinity, being 8.8% higher than that of recycled polypropylene without reinforcement. This is because hemp fibers are lignocellulosic fillers, and lignocellulosic fibers have a nucleating effect on the PP matrix, which is more noticeable with the use of PP-g-MA as compatibilizer [53,54].

The addition of 30% of short hemp fiber without any compatibilizers results in a decrease of the crystallinity, and this may be because the fiber-matrix interaction is not good enough, inhibiting the nucleating effect typical of these fibers. As mentioned above, the use of MLO as a compatibilizing agent also has a plasticizing effect, causing the free volume between the matrix chains to increase, thus decreasing the contact of the fibers with the matrix and decreasing the nucleating effect of the fibers in the matrix, which is reflected in the decrease in crystallinity [55].

**Figure 4:**
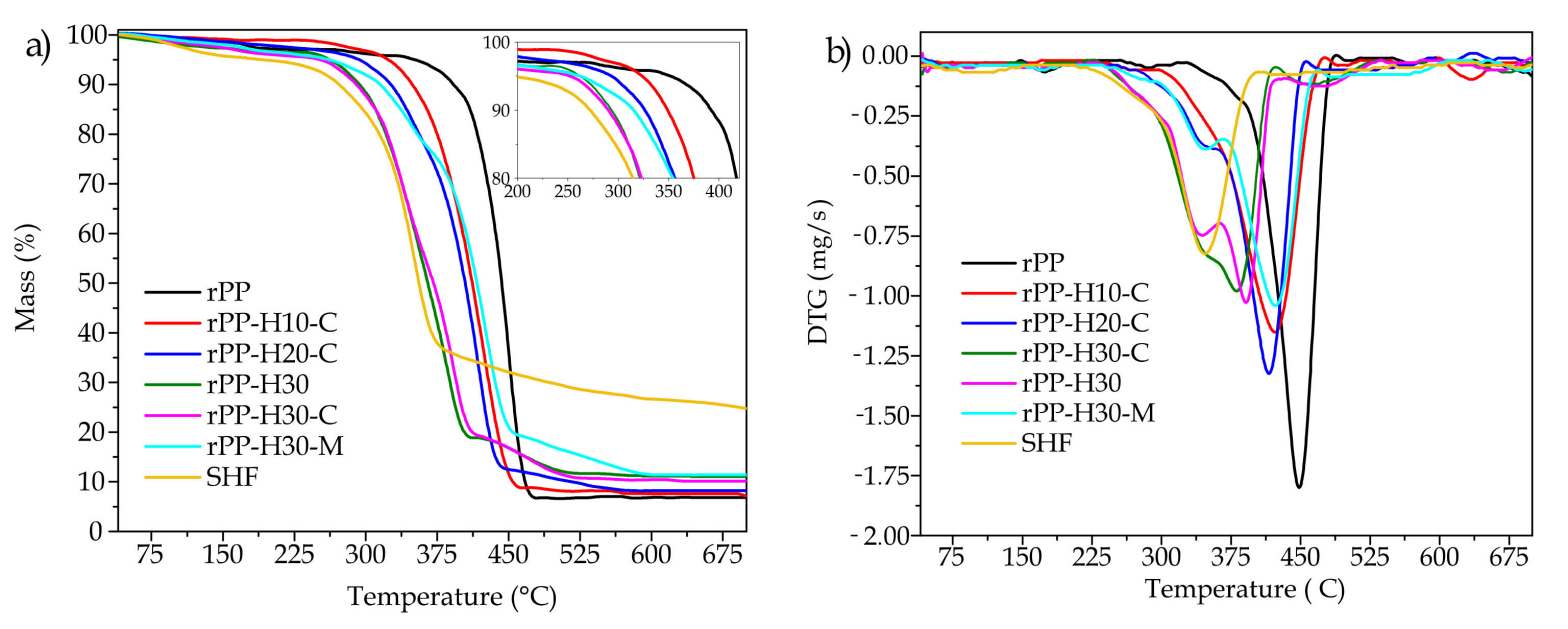
Comparative plots of (**a**) thermogravimetric curves and (**b**) DGT curves of the rPP/hemp fiber composites with different compatibilizers.

The thermal stability at high temperatures of the recycled polypropylene, the rPP/short hemp fiber composites, and short hemp fiber was determined by thermogravimetric tests. The thermogravimetric graphs of the different composites and short hemp fiber are shown in Figure 4. Table 4 summarizes the main parameters extracted from the thermogravimetric curves, i.e., onset degradation temperature (*T_5%_*) and the maximum degradation temperature (*T_deg_max_*).

It is observed that thermal degradation of short hemp fiber occurs in three main steps. The first weight loss occurs below 110 °C where the mass loss is about 3% due to the moisture content of the fibers. By increasing the temperature to ~220 °C, it is possible to observe the next degradation step, which ends at 385 °C; this corresponds to the thermal depolymerization of hemicellulose, and a mass loss of 36% was observed [56]. The last step occurs in the range between 385–600 °C, where the degradation of cellulose and lignin begins, which corresponds to the typical degradation of lignocellulosic fillers [57,58]. Moreover, it is observed that the residual mass is around 25%, consistent with the literature on typical hemp fibers [59,60]. In the case of recycled polypropylene, degradation occurs in a single stage, starting at about 350 °C and ending at 550 °C, and this behavior is typical of virgin polypropylene, as suggested by Aurrekoetxea J et al. [61]. The effect of reprocessing (recycling) on polypropylene is noticeable after the fifth reprocessing. The maximum degradation occurs at 448 °C, which corresponds to the degradation of saturated and unsaturated carbon atoms [34]. It can be noted that the incorporation of hemp fiber has a direct effect on the thermal stability of the composite materials. As the loading percentage increases, the thermal stability tends to decrease, as evidenced by the decrease in *T_5%_* and maximum degradation temperature (*T_deg_max_*), compared to the unreinforced rPP. This effect can be attributed to both the chemical structure of the hemp fiber and the action of the compatibilizing agent. Figure 4 shows that as the amount of fiber loading increases, the thermogravimetric curves of the composites have a fiber-like behavior. This is because the compatibilizing agent increases the interaction between the fiber and the matrix as the hemp fiber, having a relatively low percentage of lignin (3.7–5.7%) in its constitution, does not provide the typical thermal stability generally obtained when using lignocellulosic fillers [62], which is more noticeable at low percentages of fillers. It is important to note that by incorporating a higher percentage of fiber (i.e., 30%), the degradation of the compounds occurs in three steps. The first step is due to moisture and occurs below 115 °C. The second step occurs between 280 and 400 °C, where both the hemicellulose and cellulose parts are degraded. Between 400 and 500 °C, the polypropylene matrix and the lignin corresponding to the hemp fiber are mainly decomposed. Nevertheless, it should be underlined that all rPP/SHF-based formulations present the onset degradation temperature well above than selected processing conditions (maximum processing temperature 175 °C) and are enough thermally stable for the intended application (i.e., automotive parts, indoor or garden furniture, where the service temperature is well below the onset degradation temperature). Regarding the residual mass obtained at 700 °C, Table 4 shows that rPP has a residual mass of 6.8%. Other authors like Yuan et al. [63] reported a residual mass for PP about 0.5%; this difference can be attributed to the presence of residues inside the recycled polymer matrix and observed by FESEM (Figure 2) and have already been commented on during the microscopy analysis. Hemp degradation as commented before occurs in three steps as could be observed in Figure 4; at 700 °C, the residual mass was 25%, and similar results were obtained by Oza et al. [64]. As expected, the incorporation of the fibers inside the polymer matrix provided a progressive increment of the residual mass up to 11.3% for the rPP-H30-M.

### 3.4. Thermomechanical Properties of the Recycled Polypropylene Sample and the rPP/Hemp Fiber Composites

**Figure 5:**
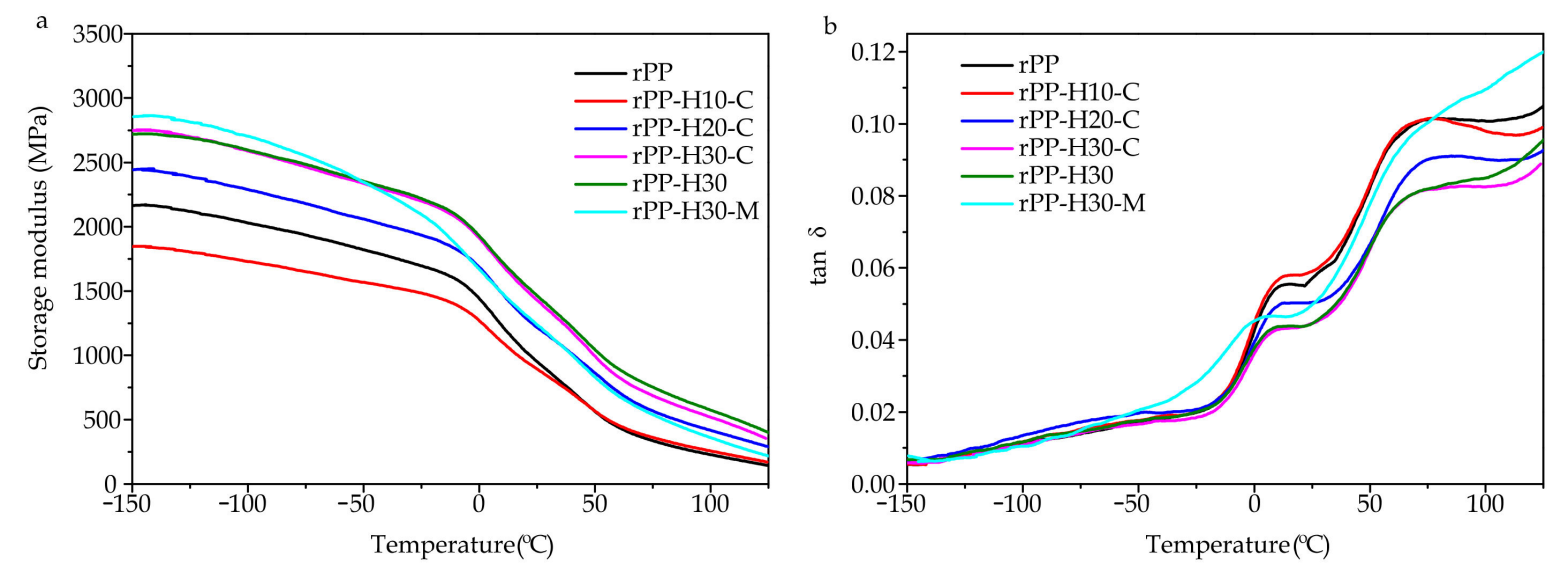
Comparative plots of the evolution of (**a**) storage modulus (E’), and (**b**) glass transition temperature (*T_g_*) as a function of the temperature of rPP pieces and the rPP/hemp fiber composites with different compatibilizers.

Figure 5 shows the comparative curves corresponding to the thermomechanical properties of the rPP sample and the rPP/hemp fiber composites. Figure 5b shows the evolution of the storage modulus (*E*’) with respect to temperature, which in general relates to the stiffness of the samples concerning the elastic energy they store. Figure 5b shows the damping factor (tan *δ*) with respect to temperature. In general, the curve presents two relaxations that are typical of polypropylene, which is observed around 14 °C, corresponding to the relaxation (*β*), being related to the glass-rubber transition of the amorphous part of the materials, which can be related to the glass transition (*T_g_*). The second relaxation occurs at around 80 °C and corresponds to relaxation (*α*), which belongs to the mechanism of lamellar sliding and rotation of the crystalline part [65]. Some of these important values are listed in Table 5.

The addition of the short hemp fiber in the recycled polypropylene matrix results in a noticeable increase in stiffness, as reflected in the increase in storage modulus values compared to the unfilled rPP sample. In addition, an increasing trend can be seen with increasing load, being more noticeable at low temperatures, these data are in agreement with those presented by Sullins et al. [36] and Etaati et al. [66]. The increase in stiffness caused by the incorporation of the hemp fiber is due to the reinforcing effect obtained with the addition of the lignocellulosic fillers, as mentioned above. This is because, with increasing fiber load, the energy absorption capacity increases, and the high interaction of the matrix with the fiber prevents energy dissipation [66].

It is worth noting a particular case of decreased stiffness that occurs with the addition of 10% hemp fiber, which is more noticeable at low temperatures. In spite of this, the reinforcing effect provided by the hemp fibers can be noticed when passing the relaxation α, reaching values slightly higher than the rPP unfilled sample. This behavior is due to the fact that the lamellar motion of the crystalline part of the polypropylene that generally occurs at this temperature is reduced by the presence of the fibers, giving a reinforcing effect to the material, which results in an increase in stiffness as suggested by John and Anadjiwala [67].

Analyzing the evolution of the damping factor as a function of temperature, it is observed that the recycled polypropylene presents a relaxation (relaxation *β*), marked by the glass transition temperature (peak), with a value of 13.5 °C, in addition to the relaxation α at a temperature of 78 °C, as mentioned above. In general, rPP/hemp composites show a slight increase in glass transition temperature; this is due to the incorporation of compatibilizing agents such as PP-g-MA, making the interaction between the matrix and the fiber good enough. In addition to this, the incorporation of fibers produces a slight decrease in the intensity of the peak damping factor; this is because the fibers restrict the mobility of the chains, causing the degree of molecular movement to decrease, thus decreasing the intensity of the damping factor [67].

The use of MLO as a compatibilizing agent results in a considerable decrease in the glass transition temperature compared to its counterpart PP-g-MA. This is due to the fact that in addition to the compatibilizing effect of the MLO, it has a plasticizing effect which generates a less dense matrix that increases the mobility of the polymeric chains and, as a consequence, decreases their *T_g_* [68]; this is in agreement with the results found in the literature [69].

### 3.5. Water Uptake Properties of the Recycled Polypropylene Sample and the rPP/Hemp Fiber Composites

**Figure 6:**
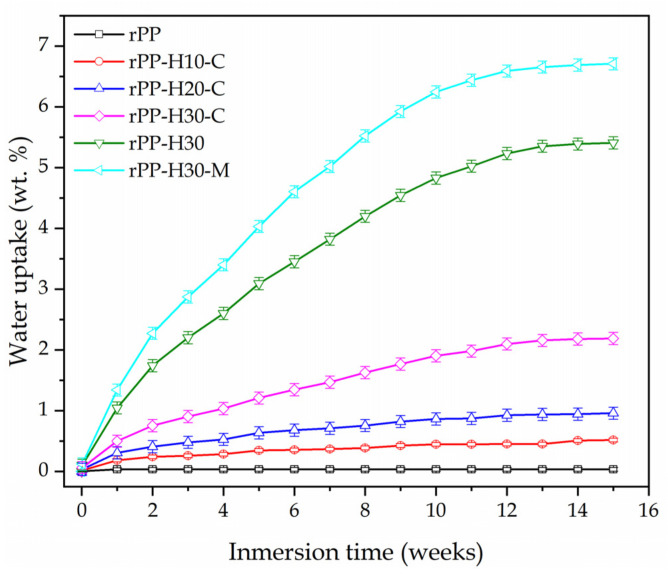
Comparative plots of the evolution of the water uptake as a function of the immersion time of rPP pieces and the rPP/hemp fiber composites with different compatibilizers.

**Scheme 1:**
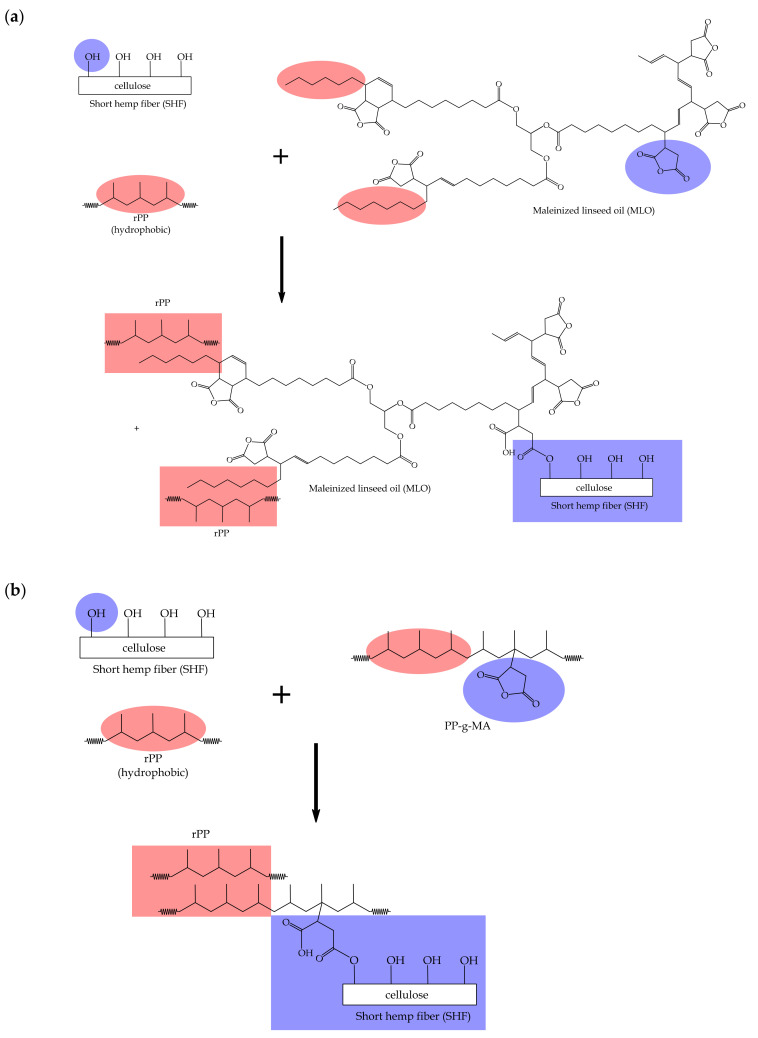
Schematic representation of compatibilization strategies of the rPP/short hemp fiber composites by the utilization of (**a**) maleinized linseed oil (MLO); (**b**) polypropylene graft maleic anhydride (PP-g-MA).

As it was already commented, the major drawback of the use of natural fibers as reinforcing fillers is their high hydrophilicity which can lead to a final hydrophilic material with poor interfacial adhesion and a tendency to decrease the overall thermomechanical performance with time. Thus, the water uptake behavior was studied. Figure 6 shows the evolution of water absorption of rPP samples and rPP/hemp fiber composites concerning soaking time (15 weeks). rPP absorbed the least amount (less than 0.2%) of water throughout the period of 15 weeks, in good agreement with the literature [70] showing the hydrophobic nature of rPP. It is observed that the addition of short hemp fiber as reinforcement has a direct impact on the water absorption capacity of composite materials. As the amount of fiber in the structure increases, the ability of the composites to absorb water tends to increase as well, mainly due to the hydrophilic nature of natural fibers as a result of the large amount of –OH groups present in hemicellulose and cellulose that tend to interact with water molecules easily [71,72]. The incorporation of PP-g-MA in the structure of the composites is noteworthy since this agent has an inhibiting effect on the water absorption capacity. Samples containing PP-g-MA and loaded with 10% fiber absorbed approximately the same amount of water as rPP samples (0.5 wt%), while samples loaded with 30% fiber absorbed only 2 wt% after 15 weeks of immersion. The optimum PP-g-MA treatment and loading made the rPP-H30-C WPC more compact by improving the interfacial adhesion between the short hemp fibers and the rPP matrix, as can be clearly seen in the FESEM image (Figure 2e). This is mainly because the maleic anhydride groups of PP-g-MA reacted with a large amount of the available –OH groups of the hemp fibers as was proposed in Scheme 1, causing the reduction of the free –OH groups in the fibers so that they are not available to react with water as suggested by Garcia-Garcia et al. [52]. This effect is clearly evident in the rPP-H30 samples which have an absorption percentage of approximately 5.5 wt%. Moreover, it is known that the roughness also has an effect on the transport properties of the porous matrix, where the equilibrium time in a single tortuous capillary with roughened surfaces increases with decreasing relative roughness [73]. As can be seen from the FESEM analysis (Figure 2), the roughness of WPCs decreased with respect to rPP. In addition, a large number of porous tubular structures existing in fiber speed up the penetration of water by the so-called capillary action [74]. Thus, with the high uncompatibilized fiber content in the rPP-H30 sample, there are highly available –OH groups which leads to highly absorbed water.

The results obtained here by compatibilizing the rPP with PP-g-MA showed better results than other compatibilizing strategies. For instance, Rachini et al. reinforced virgin PP with 30 wt% of short hemp fibers using two silane-based agents, vinyltrimethoxysilane (VTMS) and a coupling system based on VTMO and 3-(triethoxysilyl)propylsuccinic anhydride (SiAn), as compatibilizers. They observed that the water uptake of PP-SHF was increased when using VTMS as compatibilizer, and it was reduced by 43% with (VTMO + SiAn) reaching values of about 1.25% after one month of immersion in water (without reaching the steady state) [26]. Meanwhile, the rPP-H30-C developed here showed a reduction of 60% from 2.5 wt% in rPP-H30 to 1.0 wt% in rPP-H30-C after one month of immersion in water. Reference [75] studied virgin PP reinforced with 30 wt% of short hemp fibers hybridized with 10 wt% of glass fibers and observed that although the water absorption properties of the hemp fiber composites were somewhat improved by hybridization with glass fibers, the water absorption value was around 7% when the materials reach the steady state (in around five months). Meanwhile, the rPP-H30-C developed here showed a reduction of 63% (from 5.5 wt% in rPP-H30 to 2.0 wt% in rPP-H30-C) after 15 weeks of immersion in water which is the time required to reach the steady state. On the other hand, the rPP-H30-M sample presents the highest percentage of water absorption, reaching values of 7.6 wt%. This can be attributed to the plasticizing effect resulting from the use of MLO, which increases the free volume between the matrix chains, helping the diffusion of water in the composite, thus increasing the water absorption capacity [33].

### 3.6. Colorimetry Properties of rPP/Hemp Fiber Composites

One important parameter when WPCs were analyzed was the colorimetry changes when the short fiber was introduced. CIELEAB color space (L*, a*, b*) results are summarized in Table 6. The most remarkable difference between the colors appeared when 10% SHFs were introduced to rPP with a color difference ($\Delta E_{ab}^{*}$) of 23.7. The lightness (L*) of the resulting composites was reduced as a function of the SHF amount employed (87.5 for rPP and 55.0 for rPP-H30). In general, the a* values trend to red colors while the b* values trend to yellow colors in function of the amount of fiber.

**Figure 7:**
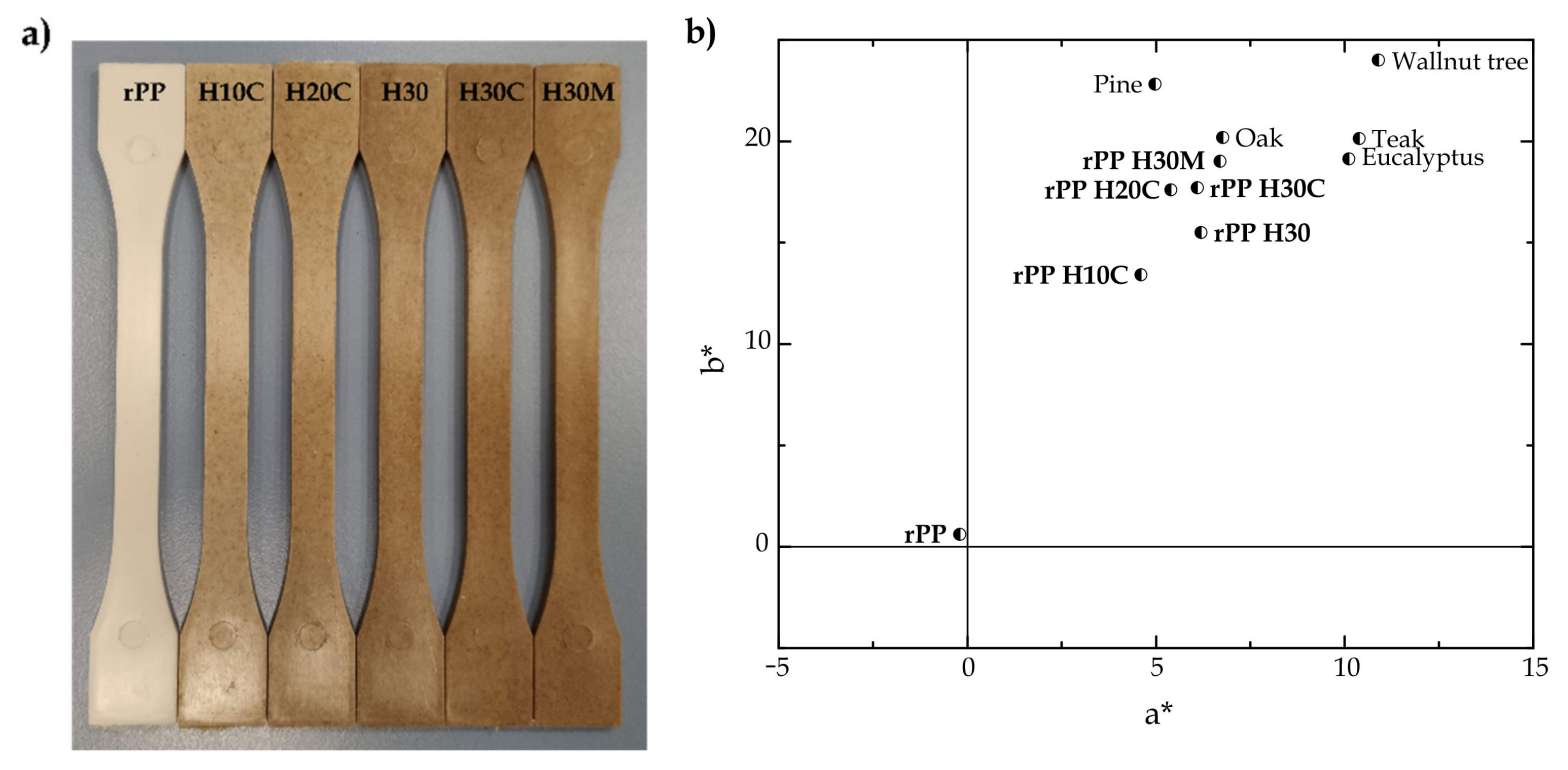
(**a**) Injection-molded rPP-SHF composites, (**b**) summary of CIELEAB color space (L*, a*, b*) results of rPP-SHF composites.

Figure 7a shows the obtained materials, and it is evident that the visual aspect significantly changed acquiring wood aspect. As can be seen in Figure 7b, the incorporation of SHF modified the color coordinates so that they acquired the appearance of woods like teak or eucalyptus [72,76,77,78]. In this instance, rPP-H30-M took an appearance very similar to oak wood. Thus, the obtained WPCs are interesting for industrial applications where wood aspect is desired such as automotive parts and construction materials (i.e., windows, frames, furniture, decking, etc.) without the need of additional additives such as dyes.

### 3.7. Chemical Structure and Infrared Spectroscopy

**Figure 8:**
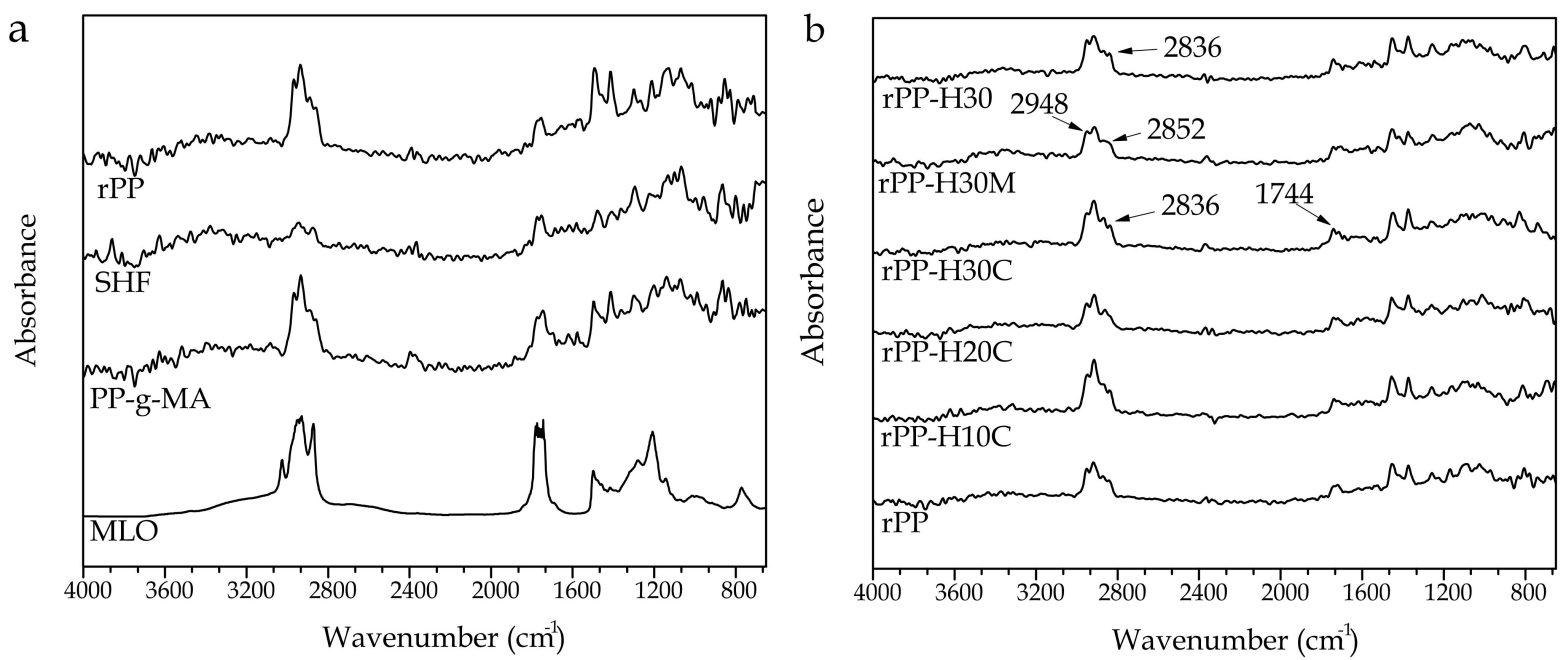
(**a**) Fourier-transform infrared (FTIR) spectra, from bottom to top, of rPP, SHF, PP-g-MA, and MLO; (**b**) FTIR spectra of the different composites.

In order to explain better the chemical composition of the green composites, the samples were analyzed by means of Fourier-transform infrared spectroscopy (FTIR). Figure 8a shows the spectrums of rPP, SHF, PP-g-MA, and MLO from 4000 to 650 cm^−1^. These values allow a better understanding of the possible interactions and chemical bonds of each of them. Furthermore, the expected chemical reaction for each compatibilization strategy is shown in Scheme 1. One of the most common processes to modify cellulose in order to improve its interaction with polymers is the esterification process. In order to carry out the process, carboxylic acids such as maleic anhydride (MAH) are used to initiate an acylation reaction [79]. In this sense, the use of MLO and PP-g-MA involves the introduction of MAH groups that interact with the OH groups of cellulose in the esterification process. In Scheme 1, the resulting structure from the esterification of the anhydride groups with the cellulose is highlighted in blue [32]. On the other hand, from the point of view of rPP in conjunction with PP-g-MA and MLO, there is a clear interaction due to the affinity between the hydrophobics of the elements, a factor that can be seen highlighted in red in Scheme 1. By using both additives, it is possible to create a bridging structure between the filler and the matrix to improve its final properties, as can be seen for example in the mechanical properties.

Regarding the polypropylene copolymer (PP-g-MA), the spectrum is very similar to that of rPP, since it is a polypropylene-based compound. However, an increase in the carbonyl band at 1785–1790 cm^−1^ characteristic of the anhydride functions can be seen [80]. In relation to the SHF, a large number of peaks appear with small intensities. This factor is due to the fiber format and the difficulty of being analyzed with the FTIR tool. In this context, a peak of pectin carboxylic groups can be seen at 1739 cm^−1^ [81]. Furthermore, a peak at 1114 cm^−1^ can also be appreciated due to the symmetric glycosidic stretching of the C–O–C bond in polysaccharide compounds of cellulose. Moreover, little absorption bands in the 1500 and 1130 cm^−1^ region can be observed, which indicate the presence of characteristic groups of cellulose, hemicellulose, and lignin. Other relevant peaks in this type of fiber are the bands of carboxylate groups at 1740 cm^−1^ and acetyl group and methyl ester at 1590 and 1240 cm^−1^ [82]. Finally, a broad absorption band is observed between 3600 and 3000 cm^−1^, which corresponds to the O–H stretching vibrations characteristic of the hydrogen bonds of the hydroxyl groups (–OH) [32]. In relation to the MLO spectrum, a first peak located at 3006 cm^−1^, corresponding to the =C–H stretching of the carbon-carbon double bonds, and those at 2925 and 2850 cm^−1^ refer to the antisymmetric and symmetric C–H stretching of the saturated carbon-carbon (C–C) bonds, respectively [32]. Furthermore, the band centered at 1735 cm^−1^ represents the carbonyl (C=O) stretching [83]. Finally, the band at 1462 cm^−1^ is due to the C–H bending vibration, while the absorption peaks at 1862 and 1784 cm^−1^ are attributed to the anhydride groups. Similar results have been reported by Jorda-Reolid et al. [84] for this type of natural compatibilizer.

Figure 8b shows the infrared spectra of all the green composites obtained. The incorporation of short hemp fibers in the rPP generates an increase in the 1744 cm^−1^ bands. This is closely related to the above-mentioned and the bonds derived from this type of natural fibers. Being more significant in the composite with a high percentage of fiber loading (30%), this band can be attributed to the esterification of the OH groups of hemicellulose and lignin and maleic groups, as mentioned above [35]. The addition of MLO in the structure of biocomposites causes a decrease in the peaks of the 2852 cm^−1^ band corresponding to the symmetrical stretching of C–H bonds, as suggested by Domici et al. [85], and the interaction between the fiber and the matrix is improved, resulting in increased mechanical properties, as discussed throughout the work. Finally, as a result of the esterification reaction between cellulose and MAH groups and the hydrophilic affinity between rPP and MLO, a green composite with good chemical affinity was generated.

## 4. Conclusions

Wood plastic composites based on a recycled polypropylene matrix reinforced with short hemp fibers were successfully obtained by injection molding. Different load-matrix compatibilization elements were analyzed, and their effect was demonstrated by the representation of the resulting chemical structures after the injection-molding process. Through this work, it was possible to determine that the recycling process to which the polypropylene textile wastes were subjected does not have a notable effect on its behavior, having practically the same characteristics as virgin polypropylene. However, it was observed that the final properties of the composites are directly related to the amount of fiber loading as well as to the quality of the interaction of the reinforcing fiber with the polymeric matrix. To improve this interaction, different compatibilizers were incorporated during manufacturing. The use of PP-g-MA as compatibilizer resulted in a remarkable improvement in the interaction of the polymeric matrix with the reinforcing fibers, as observed in the FESEM images, consequently significantly reducing the water absorption. Tensile tests indicated that increasing the amount of compatibilized short hemp fiber with PP-g-MA provides the desired reinforcing effect, which is reflected in increased stiffness as a consequence of the improvement in the interfacial strength, in addition to increasing the nucleating effect that is generally obtained by adding lignocellulosic fillers. On the other hand, the incorporation of MLO gives a plasticizing effect to the compounds, and this results in an increase of the ductile properties of the composites, improving the energy dissipated in the Charpy impact test, as well as a decrease of the glass transition temperature (T_g_). It was observed that the thermal stability is directly affected by the increase in loading, meaning that the higher the amount of hemp fiber loading, the thermal stability of the composites decreases tending to the thermal behavior of the fibers, but proving enough thermal stability for processing conditions as well as the intended use. Finally, it should be noted that the incorporation of short hemp fibers in a rPP matrix gives a color like oak wood. The best results were found for the sample compatibilized with PP-g-MA and loaded with 30 wt.% of short hemp fibers (rPP-H30-C), since this sample showed a very good interface between the matrix and the reinforcement phase, leading to an improvement of the overall thermo-mechanical performance as well as with improved hydrophobicity.

According to all these results, recycled PP textile wastes reinforced with compatibilized SHF, mainly with PP-g-MA, processed by injection molding can serve as a possible more sustainable substitute for materials currently used in several industrial applications such as automotive, construction, and furniture materials (i.e., windows, frames, decking, etc.).

## Figures and Tables

**Table 1:** Coding and formulations of the rPP/hemp fiber composites with different compatibilizers.

Code	rPP (wt%)	SHF (wt%)	PP-g-MA (phr)	MLO (phr)
rPP	100	-	-	-
rPP-H10-C	90	10	2.5	-
rPP-H20-C	80	20	5.0	-
rPP-H30-C	70	30	7.5	-
rPP-H30	70	30	-	-
rPP-H30-M	70	30	-	7.5

**Table 2:** Summary of the mechanical properties of the rPP-SHF composites, elastic modulus (*E_t_*), tensile strength (*σ_max_*), elongation at break (*ε_b_*), Shore D hardness, and impact strength.

Code	*E_t_* (MPa)	*σ_max_* (MPa)	*ε_b_* (%)	Shore D Hardness	Impact Strength (kJ m^−2^)
rPP	933 ± 45	21.2 ± 0.8	100.0 ± 11.0	70.3 ± 0.6	2.2 ± 0.1
rPP-H10-C	1617 ± 19	24.2 ± 0.7	6.7 ± 0.6	70.7 ± 0.5	2.0 ± 0.2
rPP-H20-C	2172 ± 62	26.6 ± 0.3	5.3 ± 0.6	72.7 ± 0.6	2.4 ± 0.2
rPP-H30-C	2977 ± 53	28.9 ± 0.5	5.1 ± 0.5	76.0 ± 1.0	3.2 ± 0.2
rPP-H30	2339 ± 90	22.4 ± 0.8	4.9 ± 0.7	72.8 ± 0.8	2.7 ± 0.3
rPP-H30-M	1884 ± 47	17.7 ± 0.2	8.1 ± 0.4	69.3 ± 0.6	3.7 ± 0.3

**Table 3:** Main thermal transitions obtained from DSC thermograms of the rPP/hemp fiber composites with different compatibilizers.

Code	*T_c_* (°C)	*T_m_* (°C)	Δ*H_m_* (J g^−1^)	*χ_c_max_* (%)
rPP	118.6 ± 0.6	163.7 ± 0.6	94.2 ± 0.4	68.3 ± 0.3
rPP-H10-C	117.9 ± 0.9	164.0 ± 0.6	85.2 ± 0.5	70.3 ± 0.5
rPP-H20-C	117.4 ± 0.8	163.1 ± 0.5	78.7 ± 0.5	74.9 ± 0.7
rPP-H30-C	116.8 ± 0.6	164.2 ± 0.4	66.6 ± 0.7	74.1 ± 0.4
rPP-H30	118.7 ± 0.4	165.0 ± 0.7	61.5 ± 0.6	63.7 ± 0.4
rPP-H30-M	115.7 ± 0.7	163.9 ± 0.6	64.3 ± 0.5	71.8 ± 0.5

**Table 4:** Main thermal parameters obtained by TGA of the rPP/short hemp fiber composites with different compatibilizers.

Code	*T_5%_* (°C)	*T_deg_max_* (°C)	Residual Mass (%)
rPP	352.3 ± 2.1	448.8 ± 2.3	6.8 ± 0.5
rPP-H10-C	323.8 ± 1.2	422.0 ± 1.4	7.2 ± 0.3
rPP-H20-C	292.4 ± 1.5	416.3 ± 1.6	8.2 ± 0.7
rPP-H30-C	270.3 ± 2.0	390.8 ± 1.3	11.1 ± 0.6
rPP-H30	262.3 ± 1.8	382.2 ± 1.7	10.1 ± 0.5
rPP-H30-M	261.7 ± 1.4	422.8 ± 2.2	11.3 ± 0.6
SHF	191.2 ± 1.6	345.9 ± 1.9	24.8 ± 0.4

**Table 5:** Thermomechanical parameters of rPP pieces and the rPP/hemp fiber composites with different compatibilizers in terms of storage modulus (*E*’) and glass transition temperature (*T_g_*).

Code	DMTA			
	*E*’ at −125 °C (MPa)	*E*’ at 25 °C (MPa)	*E*’ at 100 °C (MPa)	*T_g_* (°C)
rPP	2117.6 ± 50.2	951.5 ± 20.2	228.2 ± 18.2	13.6 ± 1.1
rPP-H10-C	1804.3 ± 42.4	894.6 ± 10.5	256.4 ± 16.4	14.9 ± 0.7
rPP-H20-C	2392.6 ± 33.7	1216.9 ± 25.2	417.3 ± 11.3	15.3 ± 0.6
rPP-H30-C	2695.3 ± 61.1	1428.7 ± 12.3	520.0 ± 17.4	13.6 ± 0.6
rPP-H30	2693.5 ± 64.4	1463.4 ± 30.2	573.0 ± 20.3	15.2 ± 0.9
rPP-H30-M	2818.4 ± 44.6	1239.4 ± 28.7	360.0 ± 13.1	7.6 ± 0.8

**Table 6:** Summary of the CIELEAB color coordinates of hemp fiber composites.

Code	L*	a*	b*	$\Delta E_{ab}^{*}$
rPP	87.5 ± 0.4	−0.2 ± 0.1	0.6 ± 0.2	-
rPP-H10-C	68.8 ± 0.6	4.6 ± 0.5	13.4 ± 0.5	23.7 ± 0.6
rPP-H20-C	63.8 ± 0.3	5.4 ± 0.1	17.6 ± 0.1	30.1 ± 0.1
rPP-H30-C	55.0 ± 0.2	6.2 ± 0.5	15.5 ± 0.5	36.8 ± 0.2
rPP-H30	56.9 ± 0.5	61 ± 0.4	17.7 ± 0.6	36.1 ± 0.2
rPP-H30-M	55.3 ± 0.4	6.7 ± 0.2	19.0 ± 0.4	38.2 ± 0.3

## Data Availability

Not applicable.

## References

[1] La Mantia F., Morreale M. (2011). Green composites: A brief review. Compos. Part A Appl. Sci. Manuf..

[2] Schwarzkopf M.J., Burnard M.D. (2016). Wood-plastic composites—Performance and environmental impacts. Environmental Impacts of Traditional and Innovative Forest-Based Bioproducts.

[3] Peças P., Carvalho H., Salman H., Leite M. (2018). Natural fibre composites and their applications: A review. J. Compos. Sci..

[4] Rohit K., Dixit S. (2016). A review-future aspect of natural fiber reinforced composite. Polym. Renew. Resour..

[5] Ramamoorthy S.K., Skrifvars M., Persson A. (2015). A review of natural fibers used in biocomposites: Plant, animal and regenerated cellulose fibers. Polym. Rev..

[6] Fombuena V., MD S. (2013). Study of the properties of thermoset materials derived from epoxidized soybean oil and protein fillers. J. Am. Oil Chem. Soc..

[7] Garcia D., Balart R., Sanchez L., Lopez J. (2007). Compatibility of recycled PVC/ABS blends. Effect of previous degradation. Polym. Eng. Sci..

[8] Luzi F., Fortunati E., Jimenez A., Puglia D., Pezzolla D., Gigliotti G., Kenny J.M., Chiralt A., Torre L. (2016). Production and characterization of PLA_PBS biodegradable blends reinforced with cellulose nanocrystals extracted from hemp fibres. Ind. Crop. Prod..

[9] Pawlak F., Aldas M., Parres F., Lopez-Martinez J., Patricia Arrieta M. (2020). Silane-Functionalized Sheep Wool Fibers from Dairy Industry Waste for the Development of Plasticized PLA Composites with Maleinized Linseed Oil for Injection-Molded Parts. Polymers.

[10] Chauhan V., Kärki T., Varis J. (2019). Review of natural fiber-reinforced engineering plastic composites, their applications in the transportation sector and processing techniques. J. Thermoplast. Compos. Mater..

[11] Huda M., Drzal L., Ray D., Mohanty A., Mishra M. (2008). Natural-fiber composites in the automotive sector. Properties and Performance of Natural-Fibre Composites.

[12] Luzi F., Fortunati E., Puglia D., Lavorgna M., Santulli C., Kenny J.M., Torre L. (2014). Optimized extraction of cellulose nanocrystals from pristine and carded hemp fibres. Ind. Crop. Prod..

[13] Plastic Europe Plastics—The Facts 2020. https://www.plasticseurope.org/en/resources/publications/4312-plastics-facts-2020.

[14] Drzyzga O., Prieto A. (2019). Plastic waste management, a matter for the ‘community’. Microb. Biotechnol..

[15] Li Y., Jia S., Du S., Wang Y., Lv L., Zhang J. (2018). Improved properties of recycled polypropylene by introducing the long chain branched structure through reactive extrusion. Waste Manag..

[16] Matias Á.A., Lima M.S., Pereira J., Pereira P., Barros R., Coelho J.F., Serra A.C. (2020). Use of recycled polypropylene/poly (ethylene terephthalate) blends to manufacture water pipes: An industrial scale study. Waste Manag..

[17] Gu F., Guo J., Zhang W., Summers P.A., Hall P. (2017). From waste plastics to industrial raw materials: A life cycle assessment of mechanical plastic recycling practice based on a real-world case study. Sci. Total Environ..

[18] Suaria G., Achtypi A., Perold V., Lee J.R., Pierucci A., Bornman T.G., Aliani S., Ryan P.G. (2020). Microfibers in oceanic surface waters: A global characterization. Sci. Adv..

[19] Piribauer B., Bartl A. (2019). Textile recycling processes, state of the art and current developments: A mini review. Waste Manag. Res..

[20] Yin S., Tuladhar R., Collister T., Combe M., Sivakugan N., Deng Z. (2015). Post-cracking performance of recycled polypropylene fibre in concrete. Constr. Build. Mater..

[21] Najafi S.K. (2013). Use of recycled plastics in wood plastic composites—A review. Waste Manag..

[22] Arrieta M., Fortunati E., Burgos N., Peltzer M.A., López J., Peponi L., Puglia D., Fortunati E., Kenny J.M. (2016). Nanocellulose-based polymeric blends for food packaging applications. Multifunctional Polymeric Nanocomposites Based on Cellulosic Reinforcements.

[23] Lee S.-H., Wang S. (2006). Biodegradable polymers/bamboo fiber biocomposite with bio-based coupling agent. Compos. Part A Appl. Sci. Manuf..

[24] Yatigala N.S., Bajwa D.S., Bajwa S.G. (2018). Compatibilization improves physico-mechanical properties of biodegradable biobased polymer composites. Compos. Part A Appl. Sci. Manuf..

[25] Han H., Gong X., Zhou M., Wu Y. (2020). A Study About Water/Alkali Treatments of Hemp Fiber on Ultraviolet Ageing of the Reinforced Polypropylene Composites. J. Polym. Environ..

[26] Rachini A., Mougin G., Delalande S., Charmeau J.Y., Barres C., Fleury E. (2012). Hemp fibers/polypropylene composites by reactive compounding: Improvement of physical properties promoted by selective coupling chemistry. Polym. Degrad. Stab..

[27] Wang X., Yu Z., McDonald A.G. (2019). Effect of different reinforcing fillers on properties, interfacial compatibility and weatherability of wood-plastic composites. J. Bionic Eng..

[28] Gunning M.A., Geever L.M., Killion J.A., Lyons J.G., Higginbotham C.L. (2014). Effect of compatibilizer content on the mechanical properties of bioplastic composites via hot melt extrusion. Polym. Plast. Technol. Eng..

[29] Islam M.R., Beg M.D., Gupta A. (2013). Characterization of laccase-treated kenaf fibre reinforced recycled polypropylene composites. BioResources.

[30] Srebrenkoska V., Gaceva G.B., Avella M., Errico M.E., Gentile G. (2008). Recycling of polypropylene-based eco-composites. Polym. Int..

[31] Kord B. (2013). Influence of nanoparticle on hygroscopic thickness swelling rate of composites from hemp fiber and recycled plastic. Sci. Eng. Compos. Mater..

[32] Quiles-Carrillo L., Montanes N., Sammon C., Balart R., Torres-Giner S. (2018). Compatibilization of highly sustainable polylactide/almond shell flour composites by reactive extrusion with maleinized linseed oil. Ind. Crop. Prod..

[33] Ferri J., Garcia-Garcia D., Sánchez-Nacher L., Fenollar O., Balart R. (2016). The effect of maleinized linseed oil (MLO) on mechanical performance of poly (lactic acid)-thermoplastic starch (PLA-TPS) blends. Carbohydr. Polym..

[34] Joseph P., Joseph K., Thomas S., Pillai C., Prasad V., Groeninckx G., Sarkissova M. (2003). The thermal and crystallisation studies of short sisal fibre reinforced polypropylene composites. Compos. Part A Appl. Sci. Manuf..

[35] Kim H.-S., Lee B.-H., Choi S.-W., Kim S., Kim H.-J. (2007). The effect of types of maleic anhydride-grafted polypropylene (MAPP) on the interfacial adhesion properties of bio-flour-filled polypropylene composites. Compos. Part A Appl. Sci. Manuf..

[36] Sullins T., Pillay S., Komus A., Ning H. (2017). Hemp fiber reinforced polypropylene composites: The effects of material treatments. Compos. Part B Eng..

[37] Garcia-Garcia D., Fenollar O., Fombuena V., Lopez-Martinez J., Balart R. (2017). Improvement of Mechanical Ductile Properties of Poly (3-hydroxybutyrate) by Using Vegetable Oil Derivatives. Macromol. Mater. Eng..

[38] Liminana P., Quiles-Carrillo L., Boronat T., Balart R., Montanes N. (2018). The Effect of Varying Almond Shell Flour (ASF) Loading in Composites with Poly (Butylene Succinate (PBS) Matrix Compatibilized with Maleinized Linseed Oil (MLO). Materials.

[39] Essabir H., Raji M., Laaziz S.A., Rodrique D., Bouhfid R. (2018). Thermo-mechanical performances of polypropylene biocomposites based on untreated, treated and compatibilized spent coffee grounds. Compos. Part B Eng..

[40] Ivorra-Martinez J., Manuel-Mañogil J., Boronat T., Sanchez-Nacher L., Balart R., Quiles-Carrillo L. (2020). Development and Characterization of Sustainable Composites from Bacterial Polyester Poly (3-Hydroxybutyrate-co-3-hydroxyhexanoate) and Almond Shell Flour by Reactive Extrusion with Oligomers of Lactic Acid. Polymers.

[41] Lu N., Oza S. (2013). A comparative study of the mechanical properties of hemp fiber with virgin and recycled high density polyethylene matrix. Compos. Part B Eng..

[42] Song Y.S., Lee J.T., Ji D.S., Kim M.W., Lee S.H., Youn J.R. (2012). Viscoelastic and thermal behavior of woven hemp fiber reinforced poly(lactic acid) composites. Compos. Part B Eng..

[43] Quiles-Carrillo L., Duart S., Montanes N., Torres-Giner S., Balart R. (2018). Enhancement of the mechanical and thermal properties of injection-molded polylactide parts by the addition of acrylated epoxidized soybean oil. Mater. Des..

[44] Maslinda A., Majid M.A., Ridzuan M., Afendi M., Gibson A. (2017). Effect of water absorption on the mechanical properties of hybrid interwoven cellulosic-cellulosic fibre reinforced epoxy composites. Compos. Struct..

[45] Quiles-Carrillo L., Boronat T., Montanes N., Balart R., Torres-Giner S. (2019). Injection-molded parts of fully bio-based polyamide 1010 strengthened with waste derived slate fibers pretreated with glycidyl-and amino-silane coupling agents. Polym. Test..

[46] Luyt A., Dramićanin M., Antić Ž., Djoković V. (2009). Morphology, mechanical and thermal properties of composites of polypropylene and nanostructured wollastonite filler. Polym. Test..

[47] Dolores Samper M., Bertomeu D., Patricia Arrieta M., Miguel Ferri J., Lopez-Martinez J. (2018). Interference of Biodegradable Plastics in the Polypropylene Recycling Process. Materials.

[48] Orue A., Eceiza A., Arbelaiz A. (2018). Pretreatments of natural fibers for polymer composite materials. Lignocellulosic Composite Materials.

[49] Agüero Á., Garcia-Sanoguera D., Lascano D., Rojas-Lema S., Ivorra-Martinez J., Fenollar O., Torres-Giner S. (2020). Evaluation of different compatibilization strategies to improve the performance of injection-molded green composite pieces made of polylactide reinforced with short flaxseed fibers. Polymers.

[50] Li J., Luo X., Lin X. (2013). Preparation and characterization of hollow glass microsphere reinforced poly (butylene succinate) composites. Mater. Des..

[51] Pracella M., Chionna D., Anguillesi I., Kulinski Z., Piorkowska E. (2006). Functionalization, compatibilization and properties of polypropylene composites with hemp fibres. Compos. Sci. Technol..

[52] García-García D., Carbonell A., Samper M., García-Sanoguera D., Balart R. (2015). Green composites based on polypropylene matrix and hydrophobized spend coffee ground (SCG) powder. Compos. Part B Eng..

[53] Badji C., Beigbeder J., Garay H., Bergeret A., Bénézet J.-C., Desauziers V. (2018). Natural weathering of hemp fibers reinforced polypropylene biocomposites: Relationships between visual and surface aspects, mechanical properties and microstructure based on statistical approach. Compos. Sci. Technol..

[54] Gadioli R., Morais J.A., Waldman W.R., De Paoli M.-A. (2014). The role of lignin in polypropylene composites with semi-bleached cellulose fibers: Mechanical properties and its activity as antioxidant. Polym. Degrad. Stab..

[55] Gonzalez L., Agüero A., Quiles-Carrillo L., Lascano D., Montanes N. (2019). Optimization of the loading of an environmentally friendly compatibilizer derived from linseed oil in poly(lactic acid)/diatomaceous earth composites. Materials.

[56] Rayon E., Ferrandiz S., Isabel Rico M., Lopez J., Arrieta M.P. (2015). Microstructure, Mechanical, and Thermogravimetric Characterization of Cellulosic By-Products Obtained from Biomass Seeds. Int. J. Food Prop..

[57] Yang H., Yan R., Chen H., Lee D.H., Zheng C. (2007). Characteristics of hemicellulose, cellulose and lignin pyrolysis. Fuel.

[58] Rachini A., Le Troedec M., Peyratout C., Smith A. (2009). Comparison of the thermal degradation of natural, alkali-treated and silane-treated hemp fibers under air and an inert atmosphere. J. Appl. Polym. Sci..

[59] Hussain S., Anjum F., Butt M., Sheikh M. (2008). Chemical composition and functional properties of flaxseed (*Linum usitatissimum*) flour. Sarhad J. Agric.

[60] Kabir M.M., Wang H., Lau K.T., Cardona F. (2013). Effects of chemical treatments on hemp fibre structure. Appl. Surf. Sci..

[61] Aurrekoetxea J., Sarrionandia M., Urrutibeascoa I., Maspoch M.L. (2001). Effects of recycling on the microstructure and the mechanical properties of isotactic polypropylene. J. Mater. Sci..

[62] Suardana N., Piao Y., Lim J.K. (2011). Mechanical properties of hemp fibers and hemp/pp composites: Effects of chemical surface treatment. Mater. Phys. Mech..

[63] Yuan B., Chen G., Zou Y., Shang S., Sun Y., Yu B., He S., Chen X. (2019). Alumina nanoflake-coated graphene nanohybrid as a novel flame retardant filler for polypropylene. Polym. Adv. Technol..

[64] Oza S., Wang R., Lu N. (2011). Thermal and mechanical properties of recycled high density polyethylene/hemp fiber composites. Int. J. Appl. Sci. Technol..

[65] Amash A., Zugenmaier P. (1997). Thermal and dynamic mechanical investigations on fiber-reinforced polypropylene composites. J. Appl. Polym. Sci..

[66] Etaati A., Pather S., Fang Z., Wang H. (2014). The study of fibre/matrix bond strength in short hemp polypropylene composites from dynamic mechanical analysis. Compos. Part B Eng..

[67] John M.J., Anandjiwala R.D. (2009). Chemical modification of flax reinforced polypropylene composites. Compos. Part A Appl. Sci. Manuf..

[68] Sanyang M.L., Sapuan S.M., Jawaid M., Ishak M.R., Sahari J. (2015). Effect of plasticizer type and concentration on tensile, thermal and barrier properties of biodegradable films based on sugar palm (*Arenga pinnata*) starch. Polymers.

[69] Pawlak F., Aldas M., López-Martínez J., Samper M.D. (2019). Effect of different compatibilizers on injection-molded green fiber-reinforced polymers based on poly (lactic acid)-maleinized linseed oil system and sheep wool. Polymers.

[70] Islam M.R., Rivai M., Gupta A., Beg M.D.H. (2015). Characterization of ultrasound-treated oil palm empty fruit bunch-glass fiber-recycled polypropylene hybrid composites. J. Polym. Eng..

[71] Ferrero B., Boronat T., Moriana R., Fenollar O., Balart R. (2013). Green composites based on wheat gluten matrix and posidonia oceanica waste fibers as reinforcements. Polym. Compos..

[72] Lascano D., Garcia-Garcia D., Rojas-Lema S., Quiles-Carrillo L., Balart R., Boronat T. (2020). Manufacturing and characterization of green composites with partially biobased epoxy resin and flaxseed flour wastes. Appl. Sci..

[73] Xiao B., Huang Q., Chen H., Chen X., Long G. (2021). A fractal model for capillary flow through a single tortuous capillary with roughened surfaces in fibrous porous media. Fractals.

[74] Ashori A., Sheshmani S. (2010). Hybrid composites made from recycled materials: Moisture absorption and thickness swelling behavior. Bioresour. Technol..

[75] Panthapulakkal S., Sain M. (2007). Injection-molded short hemp fiber/glass fiber-reinforced polypropylene hybrid composites—Mechanical, water absorption and thermal properties. J. Appl. Polym. Sci..

[76] Barcík Š., Gašparík M., Razumov E.Y. (2015). Effect of temperature on the color changes of wood during thermal modification. Cellul. Chem. Technol..

[77] Ostafi M.-F., Dinulică F., Nicolescu V.-N. (2016). Physical properties and structural features of common walnut (*Juglans regia* L.) wood: A case-study/Physikalische Eigenschaften und strukturelle Charakteristika des Holzes der Walnuß (*Juglans regia* L.): Eine Fallstudie. Die Bodenkult. J. Land Manag. Food Environ..

[78] Luís R.C.G., Nisgoski S., Klitzke R.J. (2018). Effect of steaming on the colorimetric properties of Eucalyptus saligna wood. Floresta Ambiente.

[79] Zhou L., Ke K., Yang M.-B., Yang W. (2020). Recent progress on chemical modification of cellulose for high mechanical-performance Poly(lactic acid)/Cellulose composite: A short review. Compos. Commun..

[80] Sclavons M., Franquinet P., Carlier V., Verfaillie G., Fallais I., Legras R., Laurent M., Thyrion F. (2000). Quantification of the maleic anhydride grafted onto polypropylene by chemical and viscosimetric titrations, and FTIR spectroscopy. Polymer.

[81] Wang B., Sain M., Oksman K. (2007). Study of structural morphology of hemp fiber from the micro to the nanoscale. Appl. Compos. Mater..

[82] Sain M., Bhatnagar A. (2003). Manufacturing of Nanofibrils from Natural Fibres, Agro Based Fibres and Root Fibres. CA Patent.

[83] Ardejani F.D., Badii K., Limaee N.Y., Shafaei S.Z., Mirhabibi A. (2008). Adsorption of Direct Red 80 dye from aqueous solution onto almond shells: Effect of pH, initial concentration and shell type. J. Hazard. Mater..

[84] Jorda-Reolid M., Gomez-Caturla J., Ivorra-Martinez J., Stefani P.M., Rojas-Lema S., Quiles-Carrillo L. (2021). Upgrading Argan Shell Wastes in Wood Plastic Composites with Biobased Polyethylene Matrix and Different Compatibilizers. Polymers.

[85] Dominici F., García García D., Fombuena V., Luzi F., Puglia D., Torre L., Balart R. (2019). Bio-polyethylene-based composites reinforced with alkali and palmitoyl chloride-treated coffee silverskin. Molecules.

